# Œdima Glottidis as a Result of Iodism

**Published:** 1890-07-05

**Authors:** 


### ŒDEMA GLOTTIDIS AS A RESULT OF IODISM.

Dr. Groenouw has collected a number of cases of œdema of the glottis setting in suddenly soon after the commencement of a course of treatment with iodide of potassium, in one of which the doses had not exceeded 0·2 grams=1½ grains! In four cases reported by Fournier tracheotomy was necessary, and two others died before the operation could be performed. In Dr. Groenouw's own case the œdema was on one side only. In several after the attack had been recovered from, much larger doses were tolerated. But the accident is frequent enough to constitute a danger to be reckoned with. Indeed, we believe this to have been the explanation of a fatal case of œdema glottidis, which came under our own notice many years ago. A "strumous" man, with scars in the neck and other evidences of the constitutional condition, a cooper by trade, who in carrying the plane in a long sweep from one end of the planks to the other was in the habit of bearing heavily on one leg, was under our care, as an out-patient, with synovitis of the knee, for which he was taking the iodide and cod-liver oil. One Sunday night, after having had a good supper, and talking cheerfully with his wife, he was suddenly seized with symptoms of suffocation, and throwing himself on his bed, was dead before medical aid could be called in. A *post-mortem* examination showed œdema of the glottis and epiglottis to be the cause, and in the light of Dr. Groenouw's collected cases we have little doubt that his was another of the same class.

